# Heavy melts, light residues: new research identifies the potential importance of Si isotope fractionation in subducting slabs

**DOI:** 10.1093/nsr/nwaf469

**Published:** 2025-10-31

**Authors:** Paul S Savage

**Affiliations:** School of Earth and Environmental Sciences, University of St Andrews, UK

During my PhD, I was involved in a paper that claimed that, as far as Si isotopes were concerned, the Earth’s mantle appeared homogeneous [1]. Of course, time makes fools of us all, and with the benefit of improved analytical accuracy and continuing sample analysis, there is now a body of research that contests our claim—including the newly published study by Liu *et al.* (2025) [2]. This new work adds to a valuable collection of papers which have allowed new insights into how the composition of Earth’s mantle may be affected by subduction zone input and processing.

The view that there should not be any major, systematic, Si isotope variability in Earth’s mantle is based on the limited degree of Si isotope fractionation recorded during mantle melting, and, the observation that the continental crust has, on average, a very similar composition to Earth’s mantle [3 and references therein]. A growing number of studies bring this into question: some ‘HIMU’-flavour ocean island basalts (OIBs) are isotopically light compared to BSE [4], as are a suite of continental arc melts [5]. The authors of both these studies cite subduction of isotopically light sedimentary material to the mantle to explain these signatures, which although important, may be volumetrically and secularly minor in terms of BSE.

What is potentially more significant for the Si isotope homogeneity of the BSE is the observation made in Liu *et al*.’s new paper [2], supporting those from a previous study [6], that partial melting of subducting crust can generate an isotopically heavy melt, leaving an isotopically light restite. In [2], these findings are based on the careful characterization and analysis of a series of mantle-derived potassic lavas (‘lamproites’) and associated mantle xenoliths from southern Tibet, coupled with petrogenetic modelling. The anomalously heavy isotope composition of these rocks is explained by the melting of a mantle source that had previously been metasomatized by a slab-derived melt—specifically that of subducting metabasalt. A similar finding was made in [6], wherein analysis of modern adakites (basaltic slab-derived melts) were also found to be isotopically heavy.

The requirement for the generation of these heavy, slab-derived melts, as highlighted by modelling [2,6], is the presence of both garnet and clinopyroxene in the restite which, under equilibrium conditions, concentrate the lighter Si isotopes [6,7]; the heavy melt may make it to the surface (e.g. in young oceanic arcs in the case of adakites) or may react and therefore generate isotopically heavy zones of sub-continental lithospheric mantle, which could then be accessed by subsequent melts (in the case of the lamproites studied in [2]).

What is, in my opinion, the most important result of this work is the identification of an isotopically light Si isotope reservoir which could be introduced to the mantle; i.e. that removal of an isotopically heavy melt from a subducting basalt would leave an isotopically light eclogitic residue. This then predicts that slab melting, which is thought to have been a widespread process throughout Earth history [e.g. 8], will generate a dense and isotopically light (with respect to the Si isotope composition of BSE) eclogite, which should sink through the mantle, and may build up at the core–mantle boundary. Over Earth history, this subduction zone processing could lead to an overall slightly isotopically heavier, more fertile, mantle containing slivers of isotopically light, relatively unreactive, physically dense material. This then offers a new way to interpret the isotopically light HIMU OIB Si data [4]: previously, these were interpreted as being due to recycled sediment, but this is difficult to explain via mass balance (and doesn’t fit with the canonical HIMU model, e.g. [9]). In contrast, recycled oceanic crust is often cited as being a source of the HIMU mantle end-member; if **some** of this crust was melt-depleted on its way down, this may explain why some (but not all) HIMU OIBs have lighter Si isotope compositions than the BSE.

With each new study, I am grateful that despite our initial claims [1], the community continues to find value and utility in Si isotopes for studying mantle processes.
